# Assessing hypoglycemia frequency using flash glucose monitoring in older Japanese patients with type 2 diabetes receiving oral hypoglycemic agents

**DOI:** 10.1111/ggi.13765

**Published:** 2019-09-04

**Authors:** Hironori Abe, Junpei Shikuma, Hirotsugu Suwanai, Koji Sano, Takako Okumura, Kenshi Kan, Tomono Takahashi, Takashi Miwa, Ryo Suzuki, Masato Odawara

**Affiliations:** ^1^ Department of Diabetes, Metabolism, Endocrinology, Rheumatology and Collagen Diseases Tokyo Medical University Tokyo Japan

**Keywords:** blood glucose fluctuation, flash glucose monitoring system, hypoglycemia, type 2 diabetes mellitus

## Abstract

**Aim:**

It is important to consider hypoglycemia for glycemic control in elderly patients with type 2 diabetes. Continuous blood glucose monitoring system is an effective method to investigate blood glucose fluctuation. This study examined hypoglycemia frequency using continuous blood glucose monitoring system in older patients with type 2 diabetes.

**Methods:**

A total of 70 patients with type 2 diabetes aged >65 years, receiving oral treatment only and having a glycated hemoglobin (HbA1c) level of <8% were enrolled. Flash glucose monitoring system was used for the device. Patients were classified into three groups according to the type of medicine administered, in addition to other oral hypoglycemics, and were compared: (i) those taking sulfonylureas (SU); (ii) those taking glinides; and (iii) those who did not take either SU or glinides.

**Results:**

There was a significant positive correlation between the coefficient of variation and hypoglycemic frequency in all the patients, and a significant negative correlation between HbA1c and hypoglycemia in those receiving SU. When hypoglycemia was defined as glucose levels <54 mg/dL and <70 mg/dL, the cut‐off HbA1c values for developing hypoglycemia were 6.3% and 6.7%, sensitivity was 75.0% and 76.2%, and specificity was 90.9% and 77.6%, respectively.

**Conclusions:**

In older patients with type 2 diabetes receiving SU, hypoglycemic frequency increases with decreases in HbA1c level. In particular, in patients with HbA1c levels of <6.3% receiving SU, it is necessary to consider medication modification. **Geriatr Gerontol Int 2019; 19: 1030–1035**.

## Introduction

With an increasing older population, Japan has been transitioning into a super‐aging society. As older individuals with diabetes tend to experience cognitive deterioration and physical dysfunction, management and evaluation of their disease is imperative. Individual selection of the optimum treatment while considering cognitive function, activities of daily living, economic situation, psychological condition and so on is necessary for achieving glycemic control. In Japan, new guidelines on the treatment of diabetes in older individuals have been formulated on the basis of the “Glycemic Targets for Elderly Patients with Diabetes.” In this guideline, older adults were classified into three categories according to cognitive function and activities of daily living, while the target glycated hemoglobin (HbA1c) was set according to the presence or absence of drugs that might cause severe hypoglycemia (insulin, sulfonylurea [SU] and glinide).^1^ Hypoglycemia, a risk factor for dementia, depression, fractures and cognitive decline in older patients with diabetes, can be recognized through self‐monitored blood glucose measurements.^2^, ^3^, ^4^, ^5^, ^6^, ^7^ In Japan, self‐monitored blood glucose measurement has only been indicated for patients with type 2 diabetes receiving insulin or glucagon‐like peptide‐1 receptor agonists, and is uncommon among those receiving only oral treatment. Thus, patients with advanced type 2 diabetes receiving only oral medication might have difficulty recognizing hypoglycemia. We carried out the present study to investigate the frequency of hypoglycemia using continuous blood glucose monitoring (CGM) and to understand unrecognized hypoglycemia in older patients with type 2 diabetes receiving oral hypoglycemic agents. In particular, the target blood glucose level differs depending on whether medications that are prone to hypoglycemia (SU and glinide) in the Japanese elderly people's practice guidelines for diabetes.^1^ In clinical practice, we focused on whether there is a difference in the incidence of hypoglycemia in the presence or absence of SU or glinides in older patients with type 2 diabetes who only take oral medication.

## Methods

The present prospective observational study included patients who visited Tokyo Medical University Hospital, Tokyo, Japan, between 1 May 2017 and 31 August 2018. All patients provided written informed consent after information regarding the study procedure and content had been provided. This study was approved by the Ethics Review Committee (No: 2017–055), and was carried out in accordance with the Declaration of Helsinki.

### *Study design*

Patients were classified into three groups depending on whether they were receiving oral medication (SU or glinides) that was highly likely to cause hypoglycemia among those aged >65 years with type 2 diabetes. In addition to other oral hypoglycemics, group S comprised those taking SU, group G comprised those taking glinides and group N comprised those taking neither SU nor glinides. The selection criteria for patients were as follows: (i) patients with an HbA1c of <8% within the past 3 months; (ii) those who had been receiving oral hypoglycemics for at least 6 months and had not altered their medication within the past 3 months; and (iii) those who agreed to participate in this study. Patients receiving insulin or glucagon‐like peptide‐1 receptor agonists were excluded. Flash glucose monitoring (FGM) was carried out for 14 consecutive days, after which blood glucose fluctuations were analyzed.

### *CGM data collection*

FreeStyle Libre Pro, an FGM device used for CGM, can measure glucose concentrations in subcutaneous interstitial fluid every minute and record the representative value every 15 min (96 times a day). The main features of this device include: (i) its inexpensiveness; (ii) its long‐term measurement capability (14 days) using one sensor; and (iii) non‐necessity for corrections during self‐monitoring of blood glucose.^8^, ^9^ In the present study, FGM devices were worn for 14 days, but blood glucose data from 12 h after wearing an FGM up to day 10 were used for analysis according to US Food and Drug Administration recommendations. Throughout the 14‐day period in which the FGM device was worn, oral hypoglycemic medications remained unchanged. Furthermore, patients were instructed to continue with their diet and exercise therapy, which had been carried out before study inclusion. Our analysis excluded data during the days the device was attached and detached, which could likely disturb the accuracy of FGM.

### *Study outcomes*

For each group, the mean blood glucose, standard deviation (SD), coefficient of variation (CV), mean of daily difference in blood glucose (MODD), hypoglycemia frequency, and hyperglycemia frequency were compared and examined. Mean was the mean of recorded blood glucose levels, SD was the SD of daily blood glucose levels, CV was the SD divided by the mean blood glucose levels and MODD was the average difference between blood glucose levels at the same time of 2 days. MODD was calculated for two consecutive days, and their average values were used for analysis. Hypoglycemia and hyperglycemia were defined as blood glucose levels <54 and ≥180 mg/dL, respectively.^10^ Hypoglycemia and hyperglycemia frequencies were defined as the proportion of the number of glucose measurements recorded that met the definition of hypoglycemia and hyperglycemia, respectively, to the total number of glucose measurement recorded by FGM. All three groups were compared according to each parameter, examining the correlation between hypoglycemic frequency and HbA1c. Patients whose blood glucose level fell to <54 mg/dL even during one of the 96 recorded instances in a day (approximately 1%) were diagnosed with hypoglycemia. We generated receiver operating characteristic (ROC) curves to calculate the predictable HbA1c critical points that cause hypoglycemia, and analyzed the cut‐off value when hypoglycemia was defined as <54 or <70 mg/dL.

### *Statistical analysis*

The sample size was calculated based on studies of CGM including insulin treatment, because there are few similar studies using CGM in people with type 2 diabetes aged >65 years receiving only oral medication.^11^, ^12^ At least 30 patients in each group were required to have a power of 80% to detect a difference in the mean hypoglycemia frequency between study groups, assuming a mean of 0.2%, an SD of 0.4%, an α‐level of 0.05 and a dropout rate of 5%.

IBM spss Statistics version 24 (IBM Corporation, Armonk, NY, USA) was used for statistical analysis. The data are presented as mean ± SD or median (25th–75th percentile). Data were analyzed with the Kolmogorov–Smirnov and Shapiro–Wilk tests to determine their distributions. Statistical significance between groups was calculated in normally distributed data using Student's *t‐*test for independent samples or a one‐way analysis of variance, and in non‐normally distributed data using Kruskal–Wallis, Mann–Whitney *U*‐test and a one‐way analysis of variance on ranks using Bonferroni corrections for multiple comparisons. In addition, the correlation between HbA1c and hypoglycemic frequency was determined using Spearman's correlation coefficient. The significance level of all statistical tests was set at 5%. To determine the cut‐off value at which HbA1c causes hypoglycemia, a ROC curve was plotted to calculate the sensitivity, specificity and area under the curve.

## Results

### *Baseline characteristics*

Of the 80 patients included in the present study, 10 dropped out. Ultimately, 70 patients with FGM data were analyzed. The baseline patient characteristics at the start of the study and parameters of each patient group are summarized in Table 1. The median age of patients was 74.0 years (range 69.0–79.0), the mean HbA1c before study was 6.9 ± 0.42% and the mean eGFR was 66.0 ± 16.4 mL/min/1.73 m^2^. Among them, 30 patients were receiving SU and 10 were receiving glinides. Regarding SU, 25 patients received glimepiride and five received gliclazide at mean doses of 0.73 ± 0.39 and 32 ± 11 mg/day, respectively. Meanwhile, regarding glinides, nine patients received mitiglinide and one received repaglinide at mean doses of 27.8 ± 4.4 and 0.75 mg/day, respectively.

**Table 1:** Baseline characteristics

	Total (*n* = 70)	S (*n* = 30)	G (*n* = 10)	N (*n* = 30)
Men/women (*n*)	50/20	19/11	7/3	24/6
Age (years)	74.0 (69.0–79.0)	74.3 ± 6.8	74.8 ± 4.5	75.5 ± 6.1
A1c before study, NGSP (%)	6.9 ± 0.42	7.0 ± 0.50	6.9 ± 0.38	6.9 ± 0.36
eGFR (mL/min/1.73 m^2^)	66.0 ± 16.4	63.2 ± 16.6	64.3 ± 17.4	69.3 ± 15.8
Height (cm)	164.0 (157.0–169.0)	161.0 ± 10.8	164.2 ± 10.0	164.5 (161.3–168.8)
Bodyweight (kg)	63.5 ± 12.1	66.3 ± 13.1	62.2 ± 9.7	61.4 ± 12.0
BMI (kg/m^2^)	24.0 ± 3.60	25.4 ± 3.3	25.4 (19.0–26.1)	23.0 ± 3.4
No. other drugs	2.0 (1.0–2.0)	2.0 (1.0–2.0)	1.0 (1.0–2.0)	1.0 (1.0–2.0)
SU (*n*)	30	†	‐	‐
Glinide (*n*)	10	‐	‡	‐
BG (*n*)	49	23	5	21
DPP4‐I (*n*)	57	26	8	23
α‐GI (*n*)	13	3	7	3
SGLT2‐I (*n*)	7	3	1	3

Data are presented as median and interquartile range unless otherwise indicated.

† A total of 25 patients received glimepiride and five received gliclazide; the mean dose was 0.73 ± 0.39 and 32 ± 11 mg/day, respectively.

‡ Nine received mitiglinide and 1 received repaglinide; the mean dose was 27.8 ± 4.4 and 0.75 mg/day, respectively. α‐GI, α‐glucosidase inhibitor; BG, biguanide; BMI, body mass index; DPP4‐i, dipeptidyl peptidase‐4 inhibitor; eGFR, estimated glomerular filtration rate; G, patients receiving other oral hypoglycemics with glinides; N, patients receiving oral hypoglycemics other than sulfonylureas or glinides; NGSP, National Glycohemoglobin Standardization Program; S, patients taking other oral hypoglycemics with sulfonylureas; SGLT2‐i, sodium–glucose transporter 2 inhibitor; SU, sulfonylurea.

### *Analysis of parameters derived from FGM*

A comparison of FGM data showed that the mean blood glucose was significantly higher in group S than in group N (*P* mg/day = 0.041). Group S had significantly higher SD values than group G (*P* = 0.005). Groups S and N had significantly higher CV than group G (*P* = 0.001 and 0.003, respectively). Group S had significantly higher MODD than group G (*P* = 0.011). Hypoglycemia was not observed in group G. Hypoglycemia frequency was not significantly different between groups S and N. Hyperglycemia frequency was significantly higher in group S than that in group N (*P* = 0.016; Table 2). The mean percentage of time when night‐time hypoglycemia was experienced (between 23.00 and 06.00 hours) was 29.4 ± 38.7% in group S and 41.7% ± 46.8% in group N, and no significant difference was observed between the two groups (*P* = 0.710; Table 2). Groups S and N showed a significant correlation between CV and hypoglycemia frequency (group S: *P* = 0.005, group N: *P* = 0.008). Groups S and N had no correlation between SD/MODD and hypoglycemia frequency (Table 3). Similarly, no correlation was observed between eGFR and hypoglycemia frequency in any group.

**Table 2:** Comparison between the three groups according to flash glucose monitoring data

	S	G	N	*P*‐value		
				S *vs* G	S *vs* N	G *vs* N
Mean (mg/dL)	151 (130–165)	148 (134–157)	134 (124–150)	0.91	0.041	0.37
SD (mg/dL)	47.1 (39.5–52.1)	36.0 (28.8–40.5)	41.2 (33.1–52.3)	0.005*	0.13	0.157
CV (%)	30.6 (28.2–33.6)	23.5 (19.8–25.1)	29.6 (25.7–34.4)	0.001**	0.74	0.003*
MODD (mg/dL)	36.1 (31.8–41.4)	27.5 (23.7–31.7)	30.0 (26.3–37.8)	0.011*	0.124	0.267
Hypoglycemia (%)†	<0.001 (0–0.260)	–	<0.001 (0–0.0260)	–	0.377	–
Hyperglycemia (%)	26.3 (12.3–36.6)	22.2 (11.7–31.2)	15.7 (8.00–23.8)	0.246	0.016*	0.916
Nocturnal incidence (between 23.00 and 06.00 hours), per hypoglycemia (%)‡	29.4 ± 38.7	–	41.7 ± 46.8	–	0.71	–

Data are presented as median and interquartile range unless otherwise indicated.

† The maximum value was 2.083 for patients taking other oral hypoglycemics with sulfonylureas (S), and 0.520 for patients receiving oral hypoglycemics other than sulfonylureas or glinides (N).

‡ The mean percentage of time in hypoglycemia at night (23:00–6:00).

α‐GI, α‐glucosidase inhibitor; BG, biguanide; DPP4‐i, dipeptidyl peptidase‐4 inhibitor; G, patients receiving other oral hypoglycemics with glinides; NGSP, National Glycohemoglobin Standardization Program; SGLT2‐i, sodium–glucose transporter 2 inhibitor; SU, sulfonylurea.

**Table 3:** Correlation between each parameter and hypoglycemia frequency

	rs	*P*‐value	
SD	S	−0.139	0.464
	G	–	–
	N	0.270	0.149
CV	S	0.499	0.005*
	G	–	–
	N	0.477	0.008*
MODD	S	−0.083	0.664
	G	–	–
	N	−0.043	0.821
eGFR	S	0.039	0.839
	G	–	–
N	−0.051	0.789	

* *P* < 0.05.

**P < 0.001.

CV, coefficient of variation; eGFR, estimated glomerular filtration rate; G, patients receiving other oral hypoglycemics with glinides; Mean, mean blood glucose; MODD, mean of daily difference in blood glucose; N, patients receiving oral hypoglycemics other than sulfonylureas or glinides; rs, •••; S, patients receiving other oral hypoglycemics with sulfonylureas; SD, standard deviation.

### *Relationship between hypoglycemia frequency and HbA1c*

**Figure 1:**
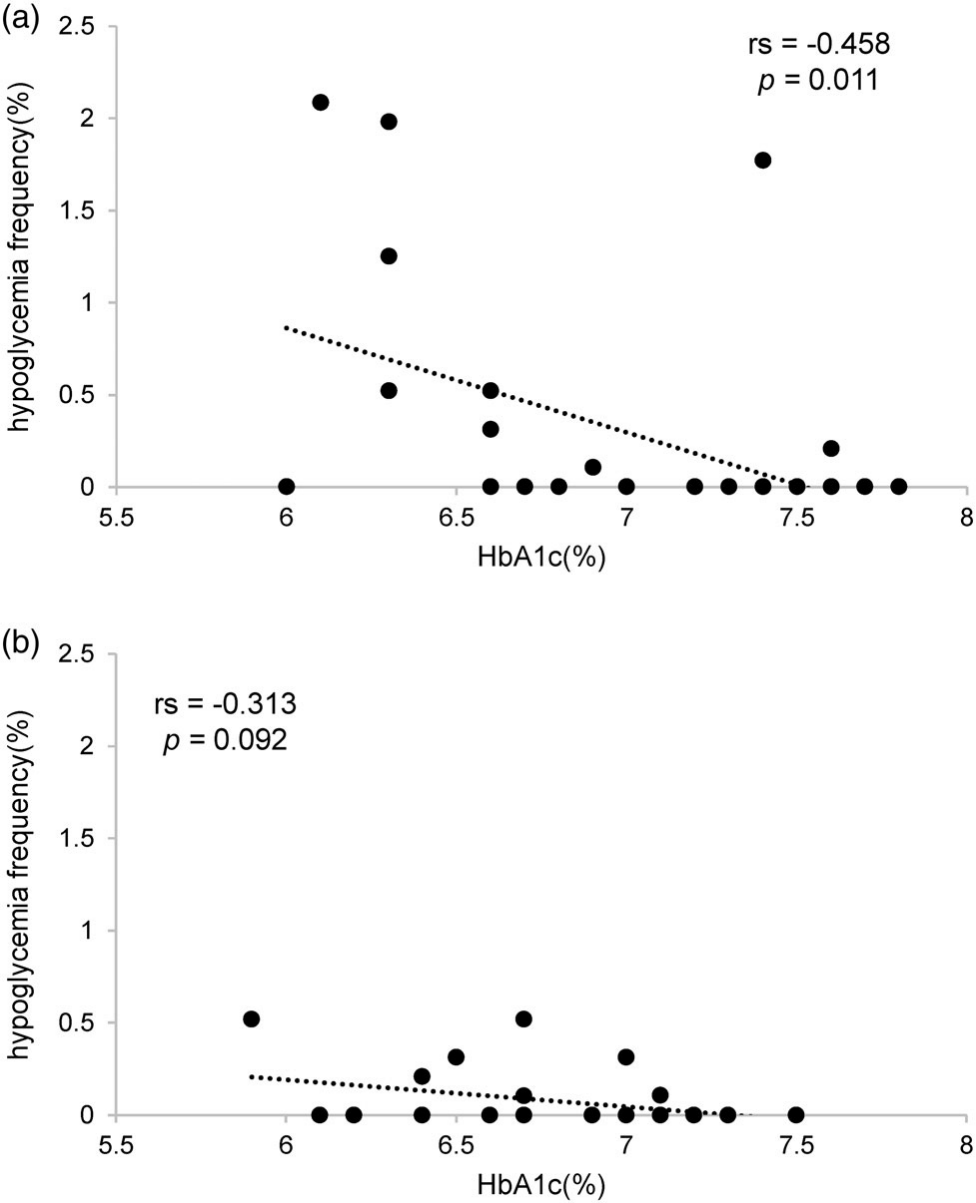
Correlation between glycated hemoglobin (HbA1c) and hypoglycemia frequency in each group. (a) Correlation between HbA1c and hypoglycemia frequency the patients taking sulfonylureas. (b) Correlation between HbA1c and hypoglycemia frequency the patients who did not take either sulfonylureas or glinides. rs, Speaman's rank correlation coefficient.

**Figure 2:**
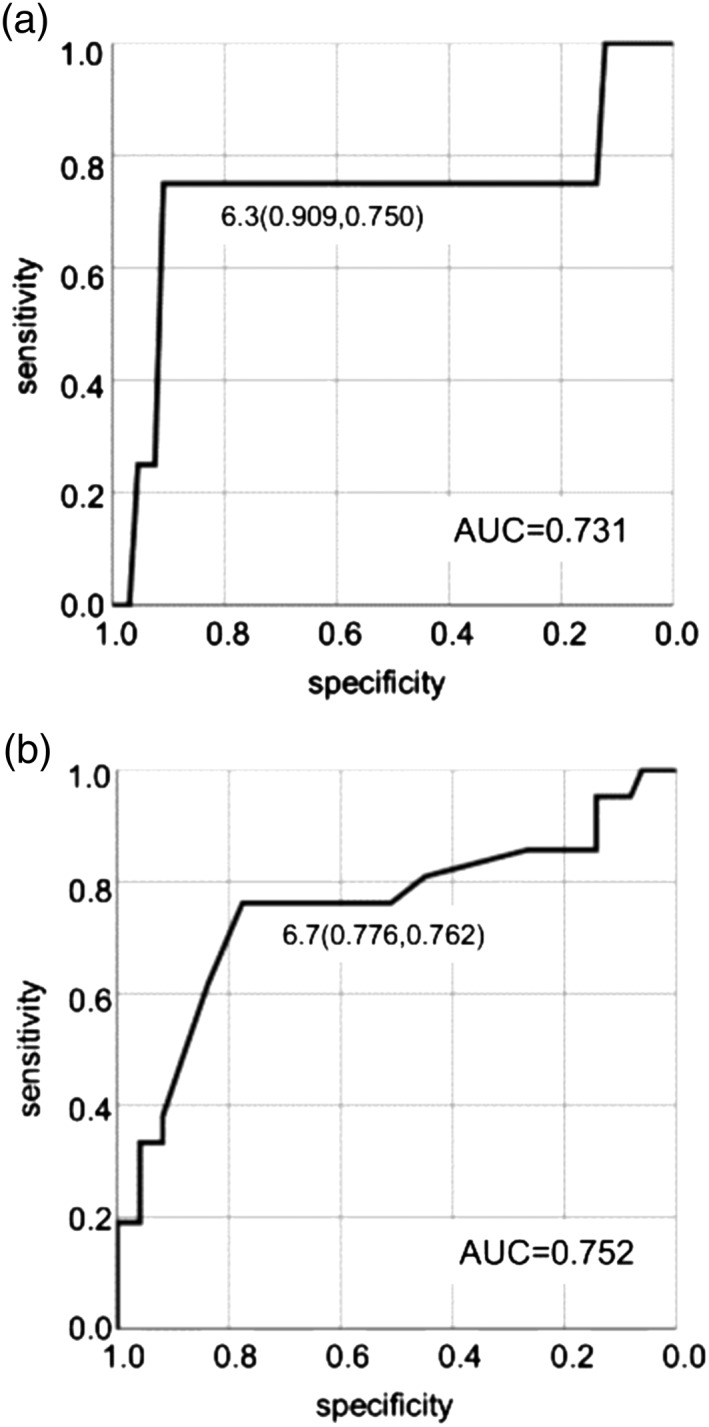
Receiver operating characteristic curves for hypoglycemia frequency and glycated hemoglobin. (a) Hypoglycemia was defined as glucose levels <54 mg/dL, the cut‐off glycated hemoglobin value for developing hypoglycemia was 6.3%, a sensitivity of 75.0% and a specificity of 90.9%. The area under the curve (AUC) was 0.731, whereas the 95% confidence interval was 0.334–1.000. (b) Hypoglycemia was defined as glucose levels <70 mg/dL, the cut‐off glycated hemoglobin value was 6.7%, a sensitivity of 76.2% and a specificity of 77.6%. The area under the curve was 0.752, whereas the 95% confidence interval was 0.609–0.895.

An inverse correlation was observed between hypoglycemia frequency and HbA1c in groups S and N. There was a significant correlation for group S, but not for group N (Fig. 1). Figure 2a represents an ROC curve for the presence of hypoglycemia and HbA1c when hypoglycemia was defined as 54 mg/dL. The cut‐off HbA1c value was 6.3%, with a sensitivity of 75.0% and specificity of 90.9%. The area under the curve was 0.731 (95% confidence interval 0.334–1.000). Additionally, when hypoglycemia was defined as glucose levels <70 mg/dL, a cut‐off HbA1c value was 6.7%, sensitivity was 76.2% and specificity was 77.6%. The area under the curve was 0.752 (95% confidence interval 0.609–0.895; Fig. 2b).

### *Analysis of differences among hypoglycemic agents*

Overall, nine patients had hypoglycemia in group S, five of whom were treated with glimepiride and four with gliclazide. The average hypoglycemia frequency in patients receiving glimepiride was 0.67 ± 0.81%, and that in patients receiving gliclazide was 1.40% ± 0.65%. There was no significant difference between the two groups (*P* = 0.190). Furthermore, seven patients had hypoglycemia in group N, of whom six (85.7%) were receiving dipeptidyl peptidase‐4 inhibitors and four (57.1%) were receiving biguanide. There were no other medications. The average hypoglycemia frequency in patients receiving dipeptidyl peptidase‐4 inhibitor in group N was 0.30 ± 0.19%, that in patients not receiving dipeptidyl peptidase‐4 inhibitor was 0.31% and there was no significant difference between the two groups. The average hypoglycemia frequency in patients receiving biguanide was 0.29 ± 0.18%, and that in patients not receiving biguanide was 0.31 ± 0.20%; there was no significant difference between the two groups.

## Discussion

The present study examined whether or not a difference in hypoglycemia frequency was present among older patients with type 2 diabetes receiving only oral hypoglycemics with and without SU and glinides. Our results showed that hypoglycemia frequency during FGM significantly increased as HbA1c decreased in group S. Although there was no significant difference, the frequency of hypoglycemia tended to be especially higher among those receiving SU. A significant correlation was found between CV and hypoglycemia frequency in groups S and N, which indicates that the hypoglycemia frequency is higher as the blood glucose fluctuation width is larger. Additionally, ROC analysis showed that the cut‐off value at which HbA1c causes hypoglycemia in older patients with type 2 diabetes receiving only oral treatment was 6.3% when hypoglycemia was defined as glucose levels <54 mg/dL, and 6.7% when hypoglycemia was defined as glucose levels <70 mg/dL. This value is roughly similar to the HbA1c lower limit indicated in the “Japanese Clinical Practice Guideline for Diabetes 2016,” further confirming the usefulness of the guideline.

According to previous reports, studies that analyzed blood glucose variability by self‐monitoring of blood glucose and CGM also point out that CV, SD, daily low blood glucose level and mean blood glucose level are better predictors of hypoglycemia than HbA1c^13^, ^14^, ^15^ Furthermore, the CV derived by FGM and the frequency of hypoglycemia showed a positive significant correlation, the large blood glucose fluctuation increased the risk of hypoglycemia in the present study. However, in actual daily outpatients, it is difficult to measure CV, SD, daily minimum blood glucose level and mean blood glucose level using CGM. If similar results can be obtained with HbA1c, which can be measured in one blood sample, the knowledge is useful for actual clinical practice. Thus, it is important to calculate the cut‐off value of HbA1c causing hypoglycemia, as in the present study. Furthermore, FGM is a relatively new device, and there are few reports of clinical trials. There have been no studies carried out specifically for Japanese patients with type 2 diabetes aged >65 years receiving only oral treatment. In this respect, the present study is considered to be novel and have usefulness in clinical practice.

Hypoglycemia has been one of the many concerning side‐effects among patients receiving insulin, SU and glinides. Hypoglycemia among older individuals can lead to falls, bone fractures, depression, arrhythmias and reduced quality of life, with severe hypoglycemia being a risk factor for dementia and cognitive decline.^5^ Although only few studies have directly compared the frequency of hypoglycemia between young and old patients with type 2 diabetes, several reports using multivariate analysis have shown that age is not a factor associated with hypoglycemia.^15^, ^16^ However, compared with younger patients with type 2 diabetes, older patients with type 2 diabetes have fewer autonomic and central nervous symptoms at the time of hypoglycemia. It is possible that a great deal of undiagnosed hypoglycemia might have appeared.^17^ Although older individuals do not have more frequent hypoglycemia, unrecognized hypoglycemia might be higher in older adults. Therefore, it is necessary to select drugs according to the appearance of hypoglycemia in older adults.

SU and glinides promote insulin secretion and exert a hypoglycemic effect through the same mechanism of action. The difference between the two drugs is that glinides have a more rapid effect with a shorter half‐life in some cases. Glinides, therefore, increase insulin secretion earlier and lower postprandial hyperglycemia. Given that its effects also subside more rapidly, fewer cases of prolonged hypoglycemia have been observed with glinides than with SU.^18^ The present study found a correlation between decreased HbA1c levels and increased hypoglycemia frequency in groups S and N, but not in group G. This phenomenon appears to be influenced by glinides' minimal influence on lowering fasting blood glucose, which promotes insulin secretion only after meals. Furthermore, a study on hypoglycemia in older Japanese individuals has reported that SU and glinides have a high odds ratio of causing hypoglycemia among the oral hypoglycemic agents. However, the hypoglycemia risk of glinides is reportedly significantly lower than that of SU.^19^ Several studies reported that repaglinide produces less hypoglycemia than SU.^20^, ^21^, ^22^ Although considering the small number of patients using glinides in the present study, it would seem that at least for older individuals, glinides are more appropriately indicated compared with SU.

In addition, the frequency of hypoglycemia was higher in group N than in group G. In previous studies, it has already been reported that hypoglycemia can occur even if SU or glinides are not administered. In the present study, one cause for the result might be that patients with hypoglycemia in group N had a lower average HbA1c level than other patients. Furthermore, we considered that the low hypoglycemia frequency found in group G is related to the effect of the small sample size or differences in background, such as insulin secretion and complications.

Although no significant difference was found, the incidence of night‐time hypoglycemia was higher in group N than in group S, although the reason for this outcome remains unclear. However, the present study was carried out on an outpatient basis without any lifestyle restrictions; therefore, it can be presumed that there is an influence of environment wherein food amount, meal time and exercise amount are not constant.

There were some limitations to this research plan. The main limitation in the present study is that the sample size was relatively small, which might affect the results of the analyses. The study registration period was limited, and the number of patients registered within the period decreased, especially in group G. Additionally, as selection bias, provided that patients who agreed to wear FGM devices had high nutritional guidance, regularly attended diabetes classes and were highly interested in their own blood sugar management, differences in adherence could be present compared with other patients. Furthermore, at the beginning of the study, we did not obtain information about diabetes complications, such as microangiopathy or macrovascular disorders, disease duration, or endogenous insulin secretion; therefore, we could not analyze the effects of these parameters on hypoglycemia. Problems with the FGM equipment itself were also present. Reports have shown several advantages for FGM, which include its non‐requirement of self‐monitoring of blood glucose for blood sugar management, its simplicity and accuracy, improved diabetes control, and discovery of unrecognized hypoglycemia.^8^, ^23^, ^24^, ^25^, ^26^ However, during FGM, differences in the displayed measurement value and the actual plasma glucose concentration might be present, particularly during rapid changes in blood glucose levels.^27^

In conclusion, the present study found a negative correlation between hypoglycemia frequency and HbA1c in older Japanese patients with type 2 diabetes receiving SU, suggesting that hypoglycemia frequency increases as HbA1c decreases. This study also determined that the cut‐off value at which HbA1c causes hypoglycemia was 6.3% when hypoglycemia was defined as glucose levels <54 mg/dL, or 6.7% when hypoglycemia was defined as glucose levels <70 mg/dL. Therefore, especially when receiving SU, once HbA1c values fall to <6.3% or <6.7%, more attention should be given to hypoglycemia, the presence of which might prompt the modification of medication.

## Disclosure statement

The authors declare no conflict of interest.
